# From Chest Trauma to Coronary Artery Dissection

**DOI:** 10.7759/cureus.61003

**Published:** 2024-05-24

**Authors:** Hakob Harutyunyan, Nnamdi Chukwuka, Arafat Ali Farooqui, Vahagn Tamazyan, Ashot Batikyan, Aleksan Khachatryan, Elliot Borgen, Joshua Kerstein

**Affiliations:** 1 Department of Internal Medicine, Maimonides Medical Center, New York, USA; 2 Department of Cardiology, Maimonides Medical Center, New York, USA; 3 Department of Internal Medicine, Washington University School of Medicine, St. Louis, USA; 4 Department of Internal Medicine, North Central Bronx Hospital, New York, USA; 5 Department of Internal Medicine, University of Maryland Medical Center, Midtown Campus, Baltimore, USA

**Keywords:** coronary computed tomography angiography, right coronary artery, coronary angiography, chest pain, blunt chest trauma, atherosclerosis, traumatic coronary artery dissection

## Abstract

Despite being rare, traumatic coronary artery dissection after blunt chest trauma can lead to life-threatening consequences that can be fatal. This case report focuses on a 51-year-old woman who suffered chest trauma at home and was later found to have right coronary artery dissection. This manuscript aims to elucidate the risk factors, diagnostic challenges, and management strategies associated with traumatic coronary artery dissection. This case report emphasizes the evaluation of risk factors, the significance of early detection with appropriate imaging modalities while maintaining high clinical suspicion, and the critical necessity of optimizing patient outcomes in such circumstances.

## Introduction

Traumatic coronary artery dissection is a rarely diagnosed complication that can arise from blunt chest trauma, potentially resulting in substantial morbidity and mortality [1]. It occurs due to the separation of the arterial wall layers, resulting in the narrowing of the coronary artery lumen, thereby causing impaired blood flow and potentially leading to myocardial ischemia or infarction. Coronary artery dissections can manifest as either asymptomatic cases or lead to acute coronary syndromes and sudden cardiac death depending on the degree of flow limitation by the dissection [2]. We present a case report of coronary artery dissection in a patient who suffered blunt chest trauma following a fall, emphasizing the risk factors, the significance of different diagnostic approaches, management strategies, and long-term follow-up.

## Case presentation

A 51-year-old female presented to the emergency department complaining of chest pain after experiencing a fall at home. Two weeks before admission, the patient slipped in the bathroom, resulting in an impact on the anterior chest wall against the sink. Since then, she had been experiencing constant chest pain radiating to the left axilla, with mild relief with ibuprofen. Additionally, she had been experiencing progressive shortness of breath. The patient denied any previous episodes of exertional chest pain but acknowledged exertional shortness of breath, which she attributed to her excessive weight. Her medical history was significant for uncontrolled type 2 diabetes mellitus, hyperlipidemia (HLD), hypertension (HTN), abdominal aortic aneurysm, two miscarriages, and polycystic ovary syndrome for which she completed a course of oral contraceptives approximately 15 years ago. The patient was taking metformin, glipizide, lisinopril, and atorvastatin at home. A physical examination revealed left anterior chest wall tenderness on palpation along with bruises.

**Figure 1:**
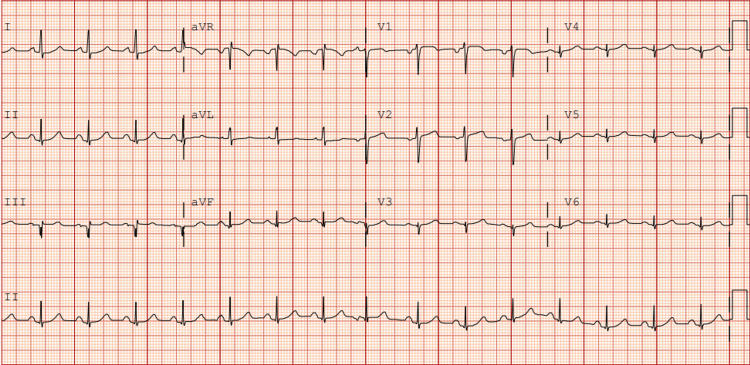
Electrocardiogram (ECG) on admission. ECG depicted in this figure demonstrates a normal sinus rhythm without ST-segment changes.

**Figure 2:**
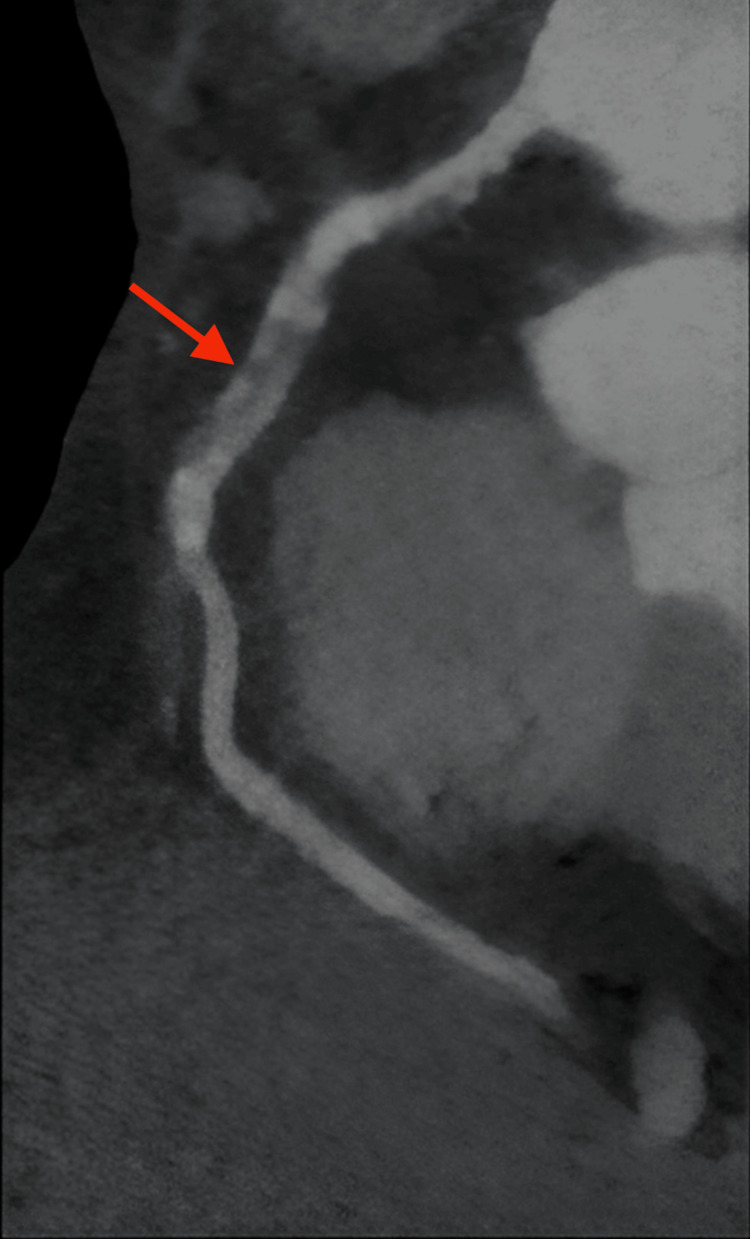
Reconstructed coronary computed tomography angiography image of the right coronary artery. The red arrow indicates a dissected segment of a proximal to middle right coronary artery with partially thrombosed false lumen.

**Figure 3:**
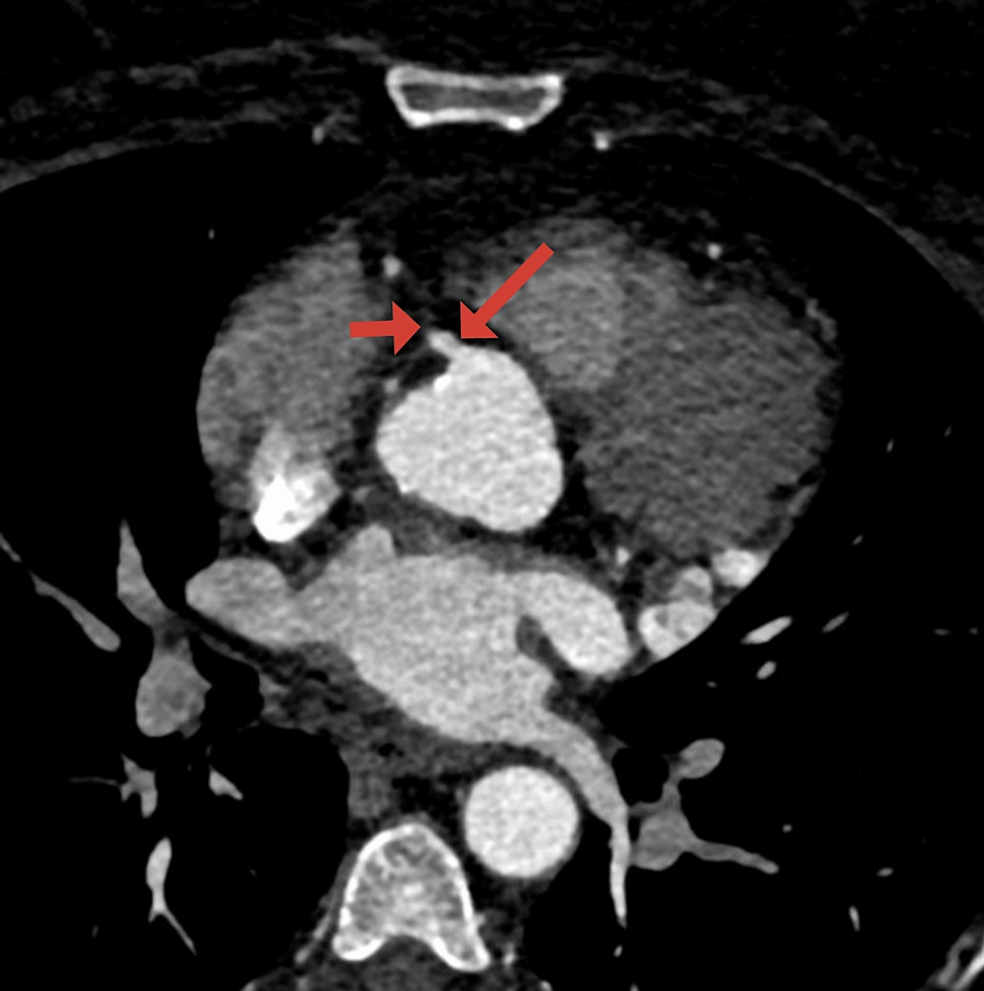
Coronary computed tomography angiography axial view of the heart. Axial view of the heart demonstrating contrast-enhanced proximal segment of the right coronary artery (long arrow) with an abrupt decrease in contrast flow in the mid-segments of the vessel (short arrow). There is no evidence of pericardial effusion.

**Figure 4:**
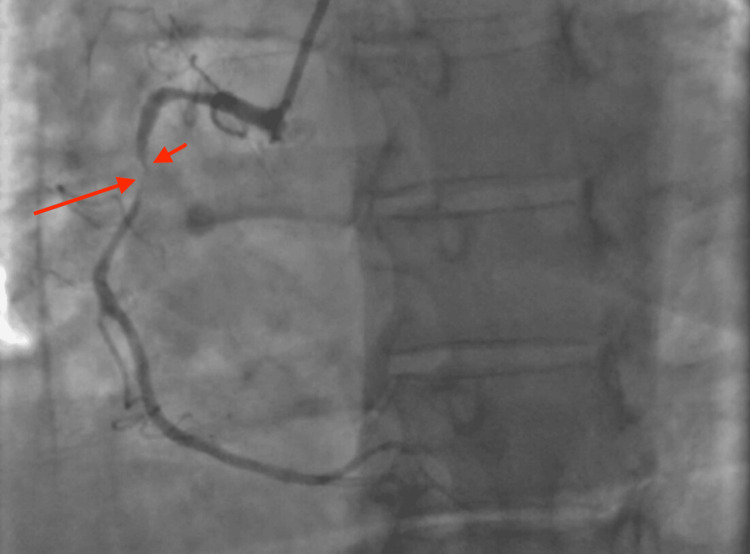
Coronary angiography of the right coronary artery. Coronary angiography of the right coronary artery demonstrates a 70% lesion in the proximal segment (short arrow), followed by a 99% narrowing in the middle segment (long arrow).

On arrival at the emergency department, the patient’s blood pressure was 154/77 mmHg, heart rate was 87 beats per minute, and oxygen saturation was 97% on room air. Initial laboratory findings were as follows: serum troponin levels of 0.05 ng/mL and then 0.03 ng/mL, blood glucose of 372 mg/dL, beta-hydroxybutyrate of 1.22 mmol/L, pH of 7.418, lactic acid of 1.4 mmol/L, anion gap of 14 mmol/L, and white blood cell count of 11.1 K/µL. Additionally, she was found to have glycated hemoglobin of 15%, low-density lipoprotein of 236 mg/dL, and positive antinuclear and ribonuclear antibodies. The electrocardiogram (ECG) was normal (Figure 1). An echocardiogram revealed an ejection fraction of 66-70% with no wall motion abnormalities. There was no evidence of bone fracture on the chest X-ray. After evaluation by the cardiology team, a coronary computed tomography angiography (CCTA) was performed, revealing a 1.5 cm long dissection in the mid-portion of the right coronary artery (RCA) with a partially thrombosed false lumen (Figure 2). There was a concern for obstructive stenosis of the true lumen along with an eccentric non-obstructive calcified plaque in the proximal aspect of the RCA. There was no evidence of pericardial effusion on axial views (Figure 3). Due to the concerns for obstructive stenosis and persistent chest pain, coronary angiography (CAG) was performed demonstrating a 70% lesion in the proximal portion, followed by a 99% lesion in the mid portion of the RCA (Figure 4).

**Figure 5:**
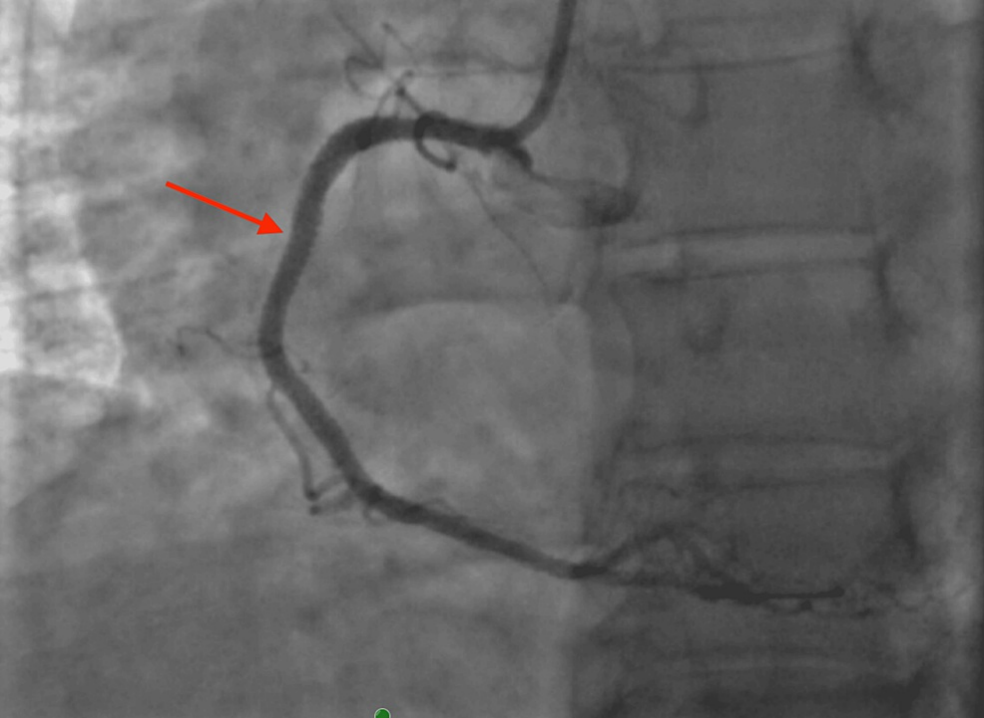
Coronary angiography of the right coronary artery. Status post-drug-eluting stent placement in the middle segment of the right coronary artery, resulting in excellent angiographic results (red arrow indicates the site of the previously narrowed segment).

A balloon angioplasty was performed, and a drug-eluting stent was successfully placed with excellent angiographic results (Figure 5).

Following the intervention, the patient was started on guideline-directed medical therapy. The patient’s chest pain resolved, and she was discharged with outpatient cardiology follow-up.

## Discussion

This case report underlines several important aspects. Primarily, it demonstrates the importance of considering coronary artery dissection as a potential cause of chest pain in patients who suffered blunt chest trauma [3]. Coronary artery dissection following blunt chest trauma is an uncommon yet highly serious complication [4]. It is important to recognize the possibility of cardiac injury not only in high-impact incidents but also in cases of low-impact trauma, as in our case, particularly in females with atherosclerosis, HTN, systemic inflammatory conditions, or with a history of hormonal therapy [3,4]. While the literature predominantly associates the above-mentioned risk factors with spontaneous coronary artery dissection (SCAD), their significance in traumatic dissection should not be overlooked. The presence of some of the above-mentioned risk factors, which are established contributors to SCAD, may have increased the patient’s susceptibility to traumatic coronary artery dissection [4].

Diagnostic challenges may arise due to overlapping symptoms with other cardiac and non-cardiac injuries. Posttraumatic musculoskeletal pain can potentially overshadow underlying angina, resulting in delayed presentation [5,6]. Prompt recognition of this condition is essential, as delayed diagnosis and management can lead to serious adverse outcomes. Therefore, it is imperative to consider the need for a comprehensive diagnostic workup in patients presenting with blunt chest trauma. According to the analysis conducted by Christensen et al., out of 77 patients with blunt chest trauma, six experienced early mortality following trauma associated with acute myocardial infarction. Among the 71 patients who survived, 90% underwent CAG, revealing that 15.8% had evidence of coronary artery dissection [5].

In our case, despite initial unremarkable ECG and echocardiogram findings, further imaging with CCTA revealed the dissection of the RCA with luminal narrowing. This highlights the complementary role of different imaging modalities in diagnosing and characterizing coronary artery dissection. While imaging studies did not indicate evidence of pericardial effusion, and the presence of a hematoma near the RCA was inconclusive, symptoms of myocardial ischemia appeared and progressed shortly after chest trauma, suggesting a causal link between the trauma and coronary issues. Furthermore, coronary angiography revealed a distinct separation of the RCA layers, consistent with dissection, localized to the proximal to mid-segment of the RCA, characteristic of a traumatic etiology [7]. Despite known risk factors for coronary artery disease, no significant atherosclerotic lesions were found, suggesting the dissection was not due to typical atherosclerosis but rather trauma. An interdisciplinary discussion concluded that dissection was most likely caused by trauma.

As per the literature review, the left anterior descending artery is the most commonly affected coronary artery in SCAD. In one review from a total of 76 CAGs, RCA dissection was detected only in 15.8% [5]. CAG is the modality of choice for diagnosing coronary artery stenosis [8]. In cases where there is uncertainty based on CAG, intracoronary imaging techniques such as optical coherence tomography or intravascular ultrasound can play a vital role in improving the accuracy of diagnosis of dissection, particularly in situations involving intramural hematoma without intimal tear. However, it is important to note that these methods come with the potential risk of causing iatrogenic dissection and therefore should not be routinely employed. In our case, CAG did not show the specific angiographic characteristics specific to intimal tear [9]. The appearance of an intramural hematoma can resemble a non-calcified atherosclerotic plaque in CCTA, thus underscoring the significance of CAG in promptly diagnosing acute cases of coronary artery dissection [10]. Conversely, in our index case, CCTA outlined the false lumen and the characteristics of the dissected wall. This argues that CCTA can be a valuable non-invasive and prompt imaging modality to use to detect coronary artery dissections. As the preferred diagnostic method for assessing significant chest trauma, the chest CT scan should consistently incorporate a thorough evaluation of the coronary arteries and myocardial perfusion using contrast-enhanced images. While a routine scan following the trauma protocol may not provide optimal visualization of the coronary arteries, the identification of perfusion defects can be valuable in indicating a potential underlying coronary injury [11]. Further research is needed to enhance our understanding of the sensitivity and specificity of CCTA in this context [12].

Management options range from conservative medical therapy to emergency percutaneous coronary intervention (PCI) or coronary artery bypass grafting, depending on the severity and location of the dissection. When a coronary artery remains open and there are no signs of ischemia, it is appropriate to opt for medical therapy. This is because previous experiences with catheter-induced dissection and angioplasty have shown that complete healing typically occurs within six months after the dissection [13]. In our case, PCI with stent placement was chosen as the preferred intervention due to persistent pain, extent, and critical nature of the dissection.

The patient had an outpatient cardiology follow-up one week post-discharge, presenting without any reported symptoms. She was compliant with her medications, including aspirin, ticagrelor, atorvastatin, metoprolol succinate, losartan, metformin, and semaglutide. No adjustments were made to her medical therapy.

## Conclusions

Our case report emphasizes the importance of considering traumatic coronary artery dissection as a potential cause of chest pain following chest wall trauma, even in low-impact incidents. It highlights the significance of CCTA in the early recognition of traumatic coronary artery dissection. It can be a valuable non-invasive and prompt imaging modality for detecting coronary artery dissections, especially in dissections without intimal tear. Further research is needed to enhance our understanding of the sensitivity and specificity of diagnostic methods, such as CCTA, and to establish the link between risk factors, such as diabetes and HLD, HTN, and coronary artery dissection following chest trauma.
